# RGSGAN–MACRNet: A More Accurate Recognition Method for Imperfect Corn Kernels Under Sample-Size-Limited Conditions

**DOI:** 10.3390/foods14244356

**Published:** 2025-12-18

**Authors:** Chenxia Wan, Wenzheng Li, Qinghui Zhang, Le Xiao, Pengtao Lv, Huiyi Zhao, Shihua Jing

**Affiliations:** 1 Key Laboratory of Grain Information Processing and Control, Ministry of Education, Henan University of Technology, Zhengzhou 450001, China; wanchenxia@haut.edu.cn (C.W.); 2023920196@stu.haut.edu.cn (W.L.);; 2 Henan Key Laboratory of Grain Photoelectric Detection and Control, Henan University of Technology, Zhengzhou 450001, China; 3 Henan Grain Big Data Analysis and Application Engineering Research Center, Henan University of Technology, Zhengzhou 450001, China; 4 Scientific Research Institute of the National Food and Material Reserve Bureau, Beijing 100037, China; 5 Inspur General Software Co., Ltd., Jinan 250101, China

**Keywords:** corn kernel imperfection recognition, data augmentation, spatial–channel synergistic attention, multi branch asymmetric convolution, generative adversarial network

## Abstract

Under sample-size-limited conditions, the recognition accuracy of imperfect corn kernels is severely degraded. To address this issue, a recognition framework that integrates a Residual Generative Spatial–Channel Synergistic Attention Generative Adversarial Network (RGSGAN) with a Multi-Scale Asymmetric Convolutional Residual Network (MACRNet) was proposed. First, residual structures and a spatial–channel synergistic attention mechanism are incorporated into the RGSGAN generator, and the Wasserstein distance with gradient penalty is integrated to produce high-quality samples and expand the dataset. On this basis, the MACRNet employs a multi-branch asymmetric convolutional residual module to perform multi-scale feature fusion, thereby substantially enhancing its ability to capture subtle textural and local structural variations in imperfect corn kernels. The experimental results demonstrated that the proposed method attains a classification accuracy of 98.813%, surpassing ResNet18, EfficientNet-v2, ConvNeXt-T, and ConvNeXt-v2 by 8.3%, 6.16%, 3.01%, and 4.09%, respectively, and outperforms the model trained on the original dataset by 5.29%. These results confirm the superior performance of the proposed approach under sample-size-limited conditions, effectively alleviating the adverse impact of data scarcity on the recognition accuracy of imperfect corn kernels.

## 1. Introduction

Corn is one of the three principal cereal crops worldwide and is widely cultivated across a diverse range of regions. It serves as an essential source of food for humans, feed for livestock and poultry, and a raw material for various industrial applications, including the production of starch, alcohol, and oil [1]. The yield and quality of corn are directly linked to food security, the development of the livestock industry, and the economic benefits of processing sectors [2,3,4,5]. In the corn quality evaluation system, kernel integrity is a crucial indicator of overall grain quality. Imperfect kernels, as a prevalent quality defect, not only impair the appearance and sensory quality of corn but may also cause the accumulation of mycotoxins, reduce nutritional value, and degrade processing performance during storage [6]. In grain trading and storage, the proportion of imperfect kernels represents a key parameter for quality grading, price assessment, and import–export inspection, with both domestic and international standards imposing strict limitations on this indicator [7,8,9,10]. Therefore, achieving efficient and accurate recognition of imperfect corn kernels is of substantial importance for ensuring grain quality and enhancing the competitiveness of the grain-processing industry.

Traditional methods for detecting imperfect kernels primarily rely on manual inspection, in which kernel color, shape, and surface texture are visually evaluated [11]. Although this approach is easy to implement, it is characterized by high labor intensity, low efficiency, intense subjectivity, and poor consistency. Some industrial sorting systems have adopted computer vision–based automatic detection, which employs machine learning and conventional image-processing algorithms for feature extraction [12,13,14,15,16,17,18,19,20]. Feature-based classification methods typically utilize color features (RGB and HSV), morphological descriptors, or texture features such as the gray-level co-occurrence matrix to construct feature vectors for SVM classification, which can achieve relatively good accuracy when the feature dimensionality is limited [21]. However, SVM-based approaches require manual feature extraction and optimization, involve complex parameter tuning, and are highly sensitive to illumination variations. Similarly, K-nearest neighbor (KNN) algorithms classify samples by voting among the K nearest neighbors in the feature space [22], but they are easily affected by noise and redundant features and cannot automatically learn feature weights. Decision tree and random forest models also rely on shape and color indices to identify imperfect kernels [23,24]; however, they tend to overfit and require manual adjustment of feature selection and the number of trees. Overall, machine learning–based methods are limited by insufficient algorithmic robustness and require extensive manual intervention. These approaches are prone to recognition errors under varying illumination, complex backgrounds, overlapping kernels, or surface contamination. In addition, they often struggle to accurately distinguish subtle textural and structural differences among different types of imperfect kernels.

In recent years, deep learning has achieved remarkable progress in image recognition [25,26,27,28,29,30,31,32]. Convolutional Neural Networks (CNNs) extract and classify features through multiple layers of convolution, pooling, and nonlinear mapping, and have demonstrated superior performance in agricultural visual recognition tasks [33,34,35,36,37,38,39,40,41,42,43]. Zhang et al. [44] proposed the LightMCS model, which integrates lightweight convolutional neural networks with self-attention mechanisms for maize seed quality recognition. With only 3.66 million parameters, the model achieved an accuracy of 84.12%. Zhang et al. [45] developed a maize variety identification approach based on hyperspectral imaging combined with an enhanced one-dimensional convolutional extended short-term memory network. To address the high model complexity and slow inference speed in maize variety identification, Zhao et al. [46] proposed an efficient and lightweight DenXt architecture. Built upon DenseNet121, the model incorporates Representative Batch Normalization and a channel attention module, achieving a classification accuracy of 97.79%. Huang et al. [47] combined hyperspectral imaging with deep learning techniques and proposed an enhanced ResNeSt_E network for detecting mechanical damage in maize kernels, achieving 99.0% accuracy in classifying four damage types. Theerthagiri et al. [48] achieved 97% classification accuracy on multiple plant disease images by incorporating a channel attention mechanism, data augmentation, and the SMOTE algorithm for dataset balancing. Dash et al. [49] achieved 94.6% accuracy in maize leaf disease identification by combining DenseNet201 deep features with Bayesian-Optimized Support Vector Machines. The incorporation of attention mechanisms has further enhanced image recognition accuracy [50,51,52]. However, deep learning models typically rely on large-scale, high-quality, and well-balanced annotated datasets [53,54,55]. For imperfect corn kernels, data availability is often constrained by seasonal harvesting and regional variability, leading to limited sample sizes and severe class imbalance. Consequently, the model performance is considerably constrained.

Data augmentation is a practical and effective approach to mitigating issues arising from small sample sizes and class imbalance. Conventional augmentation techniques, such as geometric transformations, color perturbations, and noise injection, can expand data diversity and enhance model generalization. However, these methods have limited capability in generating fine-grained defect features. They often fail to improve recognition performance under sample-size-limited conditions significantly and, in some cases, may even lead to overfitting. The advent of Generative Adversarial Networks (GANs) [56] has opened new opportunities for high-quality data generation. Through adversarial training between the generator and discriminator, GANs can learn the underlying data distribution and synthesize realistic samples. CGAN, LSGAN, and ACGAN enhance the stability of training by optimizing their respective loss functions [57,58,59]. Deep Convolutional GANs (DCGANs) [60] further improve training stability and image fidelity by adopting convolutional and transposed convolutional layers. To address instability and mode collapse, the Wasserstein GAN (WGAN) [61] introduces the Wasserstein distance to promote smoother convergence, while WGAN-GP [62] adds a gradient penalty to further enhance training stability and generation quality. In the field of agricultural visual recognition, researchers have investigated various GAN-based approaches to address data scarcity [63,64]. Zhang et al. [65] addressed the shortage of maize disease samples by proposing a mask-guided dual-perceptron generative adversarial network to synthesize complex diseased leaf images, achieving significantly higher classification accuracy after training. Zhang et al. [66] integrated hyperspectral imaging with generative adversarial networks to realize maize haploid breeding seed identification and data augmentation, yielding an average recognition accuracy improvement exceeding 10%. Zhang et al. [67] integrated near-infrared hyperspectral data with a deep convolutional generative adversarial network. In GAN-enhanced small datasets, the predictive performance of PLSR and SVR models improved significantly. Despite these advances, challenges persist in the recognition of imperfect corn kernels. On one hand, defects are often small-scale and locally distributed, making it difficult for existing generative models to capture subtle texture variations and structural features. Consequently, training instability and insufficient attention to key defect regions restrict the diversity and realism of generated samples. On the other hand, current classification networks exhibit limited capabilities for multi-scale feature fusion and texture representation under sample-size-limited conditions, hindering effective integration of local and global information and ultimately reducing recognition performance.

To address these issues, this paper proposes a sample-size-limited recognition method for imperfect corn kernels that integrates RGSGAN and MACRNet. The objective of this paper is to construct a deep learning model based on RGSGAN–MACRNet to improve the recognition accuracy of imperfect corn kernels under sample-size-limited conditions, which are common in agricultural visual recognition tasks due to the difficulty of obtaining large-scale annotated datasets. We hypothesize that introducing a spatial–channel synergistic attention mechanism in the generative stage and employing asymmetric multi-scale fusion in the classification stage can collaboratively enhance recognition accuracy. Specifically, the proposed RGSGAN incorporates residual structures and a spatial–channel synergistic attention mechanism into the generator to enhance focus on defect regions, while employing Wasserstein distance with gradient penalty to ensure training stability and sample diversity. MACRNet, on the other hand, employs multi-branch asymmetric convolutional residual networks to perform multi-scale feature fusion, reinforcing interactions between local and global representations and thereby improving both recognition accuracy and generalization. Experimental results demonstrate that the proposed method outperforms traditional DCGAN, WGAN, and Res-DCGAN in FID and IS metrics, achieving a recognition accuracy of 98.813% on the augmented dataset—an improvement of 8.3%, 6.16%, 3.01%, and 4.09% over ResNet18, EfficientNet-v2, ConvNeXt-T, and ConvNeXt-v2, respectively. Unlike existing GAN–CNN hybrids, this work introduces a novel synergy between an attention-enhanced generator and a multi-scale asymmetric convolutional classifier, achieving improvements in recognition precision under sample-size-limited conditions. The main contributions of this paper are summarized as follows:

• An RGSGAN model is designed by integrating residual structures and a spatial–channel synergistic attention mechanism into the generator, together with a Wasserstein distance and gradient penalty. This stabilizes adversarial training and enables the generation of high-quality imperfect corn kernel images under sample-size-limited conditions, thereby enhancing sample realism and diversity.
• A MACRNet model is developed, featuring a multi-branch asymmetric convolutional residual architecture for multi-scale feature fusion. This reduces parameter count and computational cost while improving the representation of fine-grained textures and global structural information.
• A hybrid data augmentation and recognition framework is proposed by combining generative and non-generative augmentation methods. This effectively alleviates data scarcity and class imbalance issues, thereby improving recognition accuracy and generalization for imperfect corn kernels.

## 2. Model and Methods

**Figure 1:**
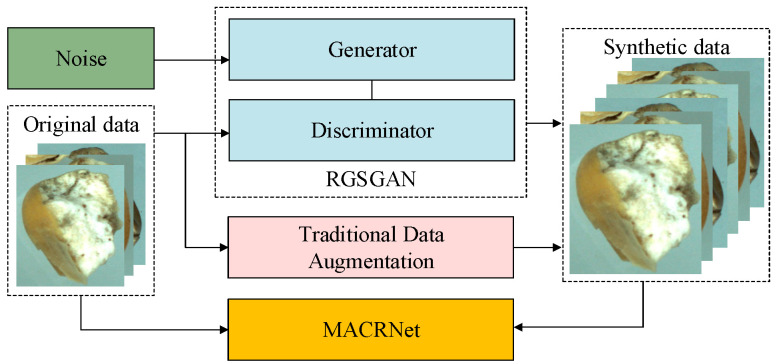
Overall architecture of the RGSGAN–MACRNet framework.

To address the low recognition accuracy of imperfect corn kernels under sample-size-limited conditions, this study proposes a two-stage recognition framework that integrates data augmentation and multi-scale feature learning. The proposed method consists of two main components: the RGSGAN and the MACRNet. The overall workflow is shown in Figure 1. In the first stage, RGSGAN introduces residual structures and a spatial–channel synergistic attention mechanism into the generator and integrates the Wasserstein distance with a gradient penalty to produce high-fidelity imperfect corn kernel images. This process effectively broadens the data distribution and enhances training stability under sample-size-limited conditions. In the second stage, MACRNet employs multi-branch asymmetric convolutional residual modules to extract and fuse multi-scale features. Through hierarchical feature learning, MACRNet strengthens the interaction between local and global information, thereby improving recognition accuracy and generalization. The overall framework synergistically enhances both data quality and feature representation, offering an efficient, scalable solution for agricultural visual recognition tasks in limited-data scenarios.

### 2.1. RGSGAN Model Architecture

**Figure 2:**
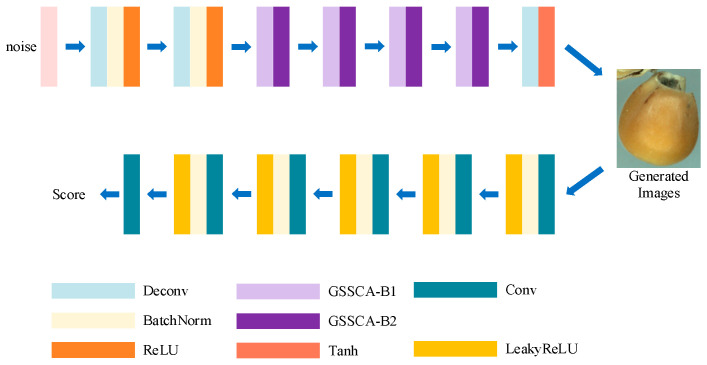
Architecture of the RGSGAN model, consisting of a generator with embedded GSCSA modules and residual connections, and a fully convolutional discriminator for adversarial training.

To improve the quality of images generated under sample-size-limited conditions, an enhanced GAN model, RGSGAN, was proposed. The overall architecture is illustrated in Figure 2. The generator takes random noise as input and performs upsampling through multiple transposed convolution layers. Based on the traditional DCGAN, the Generative Spatial–Channel Synergistic Attention (GSCSA) module is designed and used to replace part of the transposed convolution layers, enhancing the clarity and diversity of generated images. All layers of the generator adopt the ReLU activation function, except the output layer, which uses Tanh for normalization. The discriminator, in contrast, comprises six downsampling convolutional layers for feature extraction. Each layer uses a 3 × 3 convolutional kernel, followed by Batch Normalization and a LeakyReLU (0.2) activation to stabilize training. To reduce computational cost, fully connected layers are removed from the discriminator design. Through these improvements, RGSGAN effectively captures complex data features, ensuring stable training and high-quality image generation.

**Figure 3:**
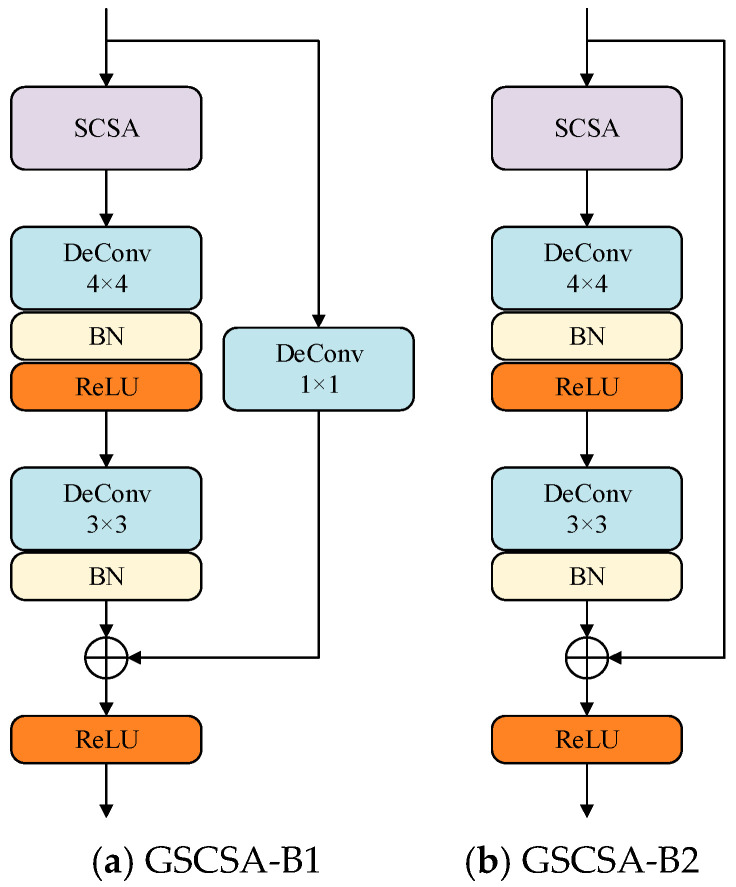
Structure of the GSCSA module.

Imperfect corn kernel defects typically appear as fine-grained, localized patterns. To enhance the generator’s focus on these regions, a GSCSA module is embedded to replace conventional upsampling layers. The GSCSA structure, shown in Figure 3, consists of two residual attention blocks (upsampling block and feature extraction block), each containing one Spatial–Channel Synergistic Attention (SCSA) module. Each block is followed by a transposed convolutional layer and a batch normalization layer, enabling efficient spatial–channel feature interaction. This design enhances feature propagation and gradient flow, mitigating the risk of vanishing or exploding gradients, and thus improving the visual clarity and structural stability of generated images.

**Figure 4:**
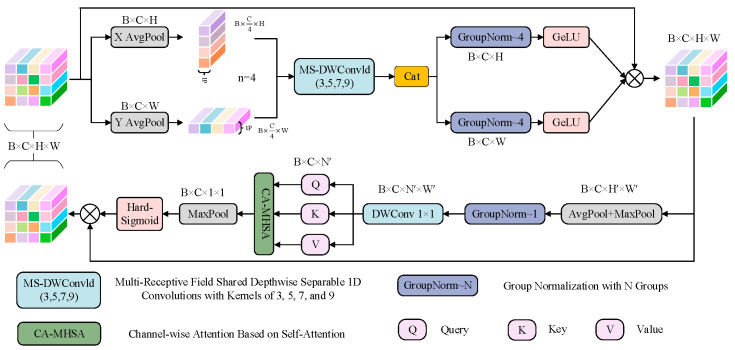
Architecture of the SCSA module, leveraging multi-semantic spatial information to steer the modeling of channel-level self-attention. In this figure, *B* indicates the batch size, *C* is the number of channels, while *H* and *W* denote the spatial dimensions of the feature maps. The symbol *n* denotes the grouping number of subdivided features, and 1*P* represents one pixel.

The SCSA module, the core component of the GSCSA module, is illustrated in Figure 4. It consists of two submodules: Semantic Multi-Scale Attention (SMSA) and Progressive Channel Self-Attention (PCSA). SMSA integrates multi-semantic information and adopts a progressive compression strategy to inject discriminative spatial priors into the channel self-attention mechanism of PCSA, thereby enabling adaptive channel recalibration. PCSA enhances inter-feature correlation through a self-attention mechanism, reducing information disparity among semantic sub-features. Along the channel dimension, SCSA employs 1D convolution to model inter-channel dependencies and adaptively emphasize channels that contribute most to classification. Along the spatial dimension, 2D convolution captures local spatial distribution features, strengthening the model’s response to defect regions. Finally, SCSA fuses spatial and channel attention weights through a synergistic learning mechanism, balancing global structural integrity with local defect precision. As a result, the generator produces images with improved overall realism and detailed fidelity.

### 2.2. Loss Function Design for RGSGAN

Traditional GAN models typically employ Jensen-Shannon divergence (JS) and Kullback–Leibler divergence (KL) to assess the disparity between the distributions of real and generated data. However, when the two distributions do not overlap, JS and KL divergences fail to provide effective gradients, leading to unstable model training. To address this, this paper introduces the Wasserstein distance with a gradient penalty term to replace the JS divergence. This enhances the generator’s ability to produce more realistic images. The Wasserstein distance is defined as:

(1) $$W\left(P_{r},P_{g}\right)=\underset{\gamma \in \Pi \left(P_{r},P_{g}\right)}{inf}\;E_{\left(x,y\right)∼\gamma }\left[∥x-y∥\right]$$

Direct computation of the Wasserstein distance proves exceedingly difficult. Consequently, WGAN employs Kantorovich-Rubinstein duality theory to derive an optimizable loss function. Its form is as follows:

(2) $$W\left(P_{r},P_{g}\right)=\underset{G}{min}\underset{D\in D}{max}\;E_{x∼p_{r}}\left[D\left(x\right)\right]-E_{z∼p_{z}}\left[D\left(G\left(z\right)\right)\right]$$

Following the introduction of the Wasserstein distance to replace the original loss function, the loss functions for the generative network and the discriminative network are as follows:

(3) $$L_{D}=-E_{x∼P_{r}}\left[D\left(x\right)\right]+E_{z∼P_{z}}\left[D\left(G\left(z\right)\right)\right]$$

(4) $$L_{G}=-E_{z∼P_{z}}\left[D\left(G\left(z\right)\right)\right]$$

Since WGAN employs weight clipping to ensure that D is 1-Lipschitz, this clipping introduces instability during training. Consequently, WGAN-GP incorporates a gradient penalty alongside the Wasserstein distance to better approximate the Lipschitz constraint. The loss functions for the discriminator and generator in WGAN-GP are as follows:

(5) $$L_{D}=E_{x∼P_{r}}[-D(x)]+E_{z∼P_{z}}[D(G(z))]+\lambda E_{\hat{x}∼P_{\hat{x}}}[\left(∥\nabla_{\hat{x}}D\left(\hat{x}\right){∥}_{2}-1{)}^{2}\right]$$

(6) $$L_{G}=-E_{z∼P_{z}}\left[D\left(G\left(z\right)\right)\right]$$

where $P_{r}$ represents the real data distribution, $P_{z}$ represents the generated data distribution, λ denotes the coefficient of the gradient penalty term, and $P_{\hat{x}}$ corresponds to the random interpolation distribution between real and generated data. By replacing the original divergence-based loss with the Wasserstein distance and replacing weight clipping with a gradient-penalized Lipschitz constraint, RGSGAN achieves more robust, stable training. This modification substantially enhances the generator’s capacity to learn the true data distribution and produce more realistic images, while the discriminator remains stable throughout the adversarial learning process, avoiding collapse.

### 2.3. MACRNet Model Architecture

**Figure 5:**
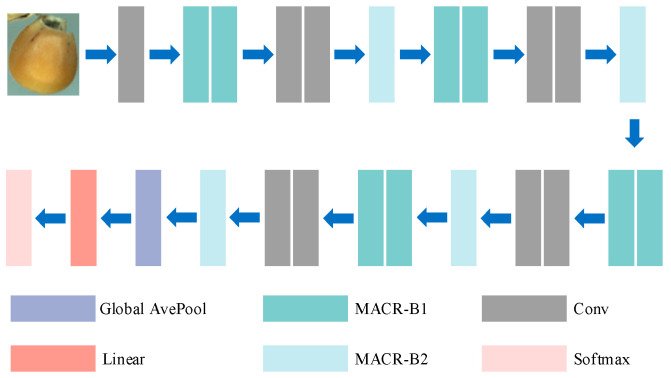
Architecture of the MACRNet model, a hierarchical residual network with multi-scale asymmetric convolutional residual modules for feature extraction and classification.

To address the difficulty of capturing local features in sample-size-limited corn kernel recognition, this paper designs a novel MACRNet, as illustrated in Figure 5. MACRNet adopts a hierarchical residual structure that progressively reduces spatial resolution and increases channel depth through downsampling modules, thereby preserving low-level details while integrating high-level semantic information. This design facilitates stable gradient propagation and deep feature learning. Compared with traditional CNNs, MACRNet’s multi-branch asymmetric convolutional structure enables simultaneous perception of local and global features, thereby enhancing the model’s representation of corn surface texture, morphological variation, and defect characteristics. The network consists of a stem layer, four feature extraction stages, and a classification head. Given an input image of size 224 × 224 × 3, the model first applies a 7 × 7 convolution (stride = 2) to obtain an initial feature map of 112 × 112 × 64. The four subsequent stages each include residual blocks (of the maintaining, convolutional, and downsampling types) for spatial compression and channel expansion. Finally, global average pooling (GAP) compresses features to a 1 × 1 × 1024 vector, which is passed to a fully connected layer for classification. This design achieves high recognition accuracy while maintaining a low parameter count and reducing the risk of overfitting.

**Figure 6:**
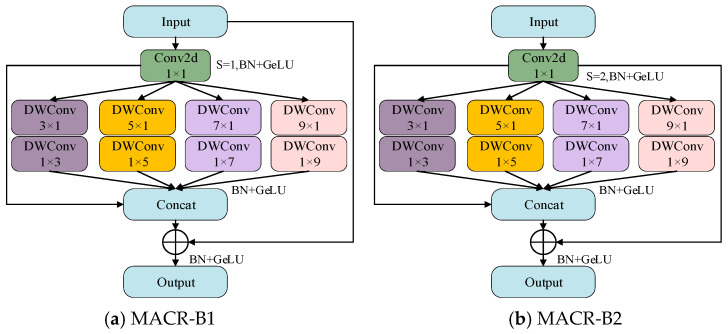
Structure of the MACR module.

The core component of MACRNet is the Multi-Scale Asymmetric Convolutional Residual (MACR) module, shown in Figure 6. Each MACR module contains five parallel convolutional branches with different kernel configurations to enable multi-scale feature perception. A 1 × 1 convolution branch performs channel mapping and feature compression. In contrast, four asymmetric convolution branches combine n × 1 and 1 × n convolutions to achieve large receptive fields with fewer parameters and lower computational cost compared with traditional large kernels. Each branch captures complementary information. From the perspective of receptive field theory, the effective receptive field of a convolutional layer expands nonlinearly with kernel size and network depth. Smaller kernels provide fine local feature extraction with strong sensitivity to high-frequency details, while larger kernels cover broader spatial contexts and capture low-frequency structural patterns. The 3 × 1/1 × 3 branch focuses on fine-grained textures such as mold spots and micro-cracks, while the 5 × 1/1 × 5 branch extracts short-range contextual relationships among adjacent surface patterns. The 7 × 1/1 × 7 branch aggregates mid-range structural features, including elongated scratches and extended stains, and the 9 × 1/1 × 9 branch models coarse global context, assisting the network in distinguishing regional defects from background variations. From a frequency-domain perspective, smaller kernels correspond to high-frequency responses that effectively capture subtle texture details, whereas larger kernels emphasize low-frequency components associated with global shape and color distributions. This multi-scale design enables the model to jointly learn and integrate local and global representations, thereby enhancing its robustness to noise, illumination variations, and intra-class diversity. Moreover, combining features of different receptive field sizes enhances both semantic abstraction and localization precision. All convolutions are implemented in a depth-wise separable manner, maintaining efficiency while preserving representational richness. These branches are concatenated along the channel dimension, followed by Batch Normalization and the GELU activation function, and then added to the residual input. Two types of MACR blocks are designed: block1 (identical input–output dimensions) and block2 (spatial downsampling with doubled channel count). Their combination ensures robust hierarchical feature representation, stable gradient flow, and mitigates network degradation during deep training. Thus, the MACR module forms the core structural unit of MACRNet, enabling efficient multi-scale learning and high-performance classification of imperfect corn kernels.

## 3. Results and Analysis

### 3.1. Dataset Construction

**Figure 7:**
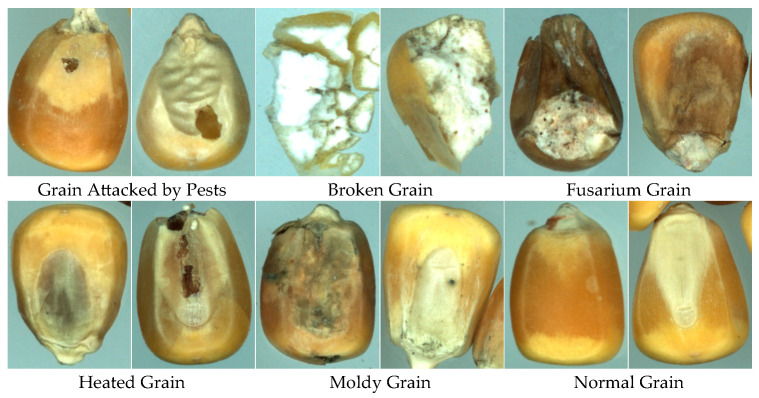
Imperfect corn kernel samples from the GrainSpace dataset.

This study employed the GrainSpace [68] dataset, collected using a G600 high-resolution camera to capture corn kernel samples. The image size is 224 × 224 × 3. The dataset contains six categories: regular kernels and five types of imperfect kernels (Grain Attacked by Pests, AP; Broken Grain, BN; Fusarium Grain, FM; Heated Grain, HD; Moldy Grain, MY; Normal Grain, NOR), with 1000 images per class, for a total of 6000 samples. In most agricultural fine-grained recognition studies, large-scale datasets are typically required for deep learning models to sufficiently learn discriminative category features. For instance, the dataset used in Paper [46] contains 31,000 images, while Paper [44] utilizes a dataset with 17,800 images spanning 4 categories. Although the dataset contains 6000 images, it still faces significant challenges in supporting high-performance corn kernel recognition. Although the dataset contains 6000 images, each class exhibits considerable intra-class variability due to inconsistent illumination, variations in kernel shape, and differing degrees of damage. Moreover, the defect categories present highly unbalanced intra-class distributions and subtle inter-class visual differences, which substantially increase the recognition difficulty. The test accuracy on 6000 samples is approximately 6% lower than that on a larger auxiliary dataset (12,000 samples). Moreover, ResNet18 and EfficientNet-v2 exhibit severe overfitting when trained on 6000 samples. Such complexity renders 1000 images per class insufficient to ensure the generalization capability of deep networks, thereby constituting a sample-size-limited condition. Representative samples are shown in Figure 7.

### 3.2. Experimental Environment and Parameter Settings

All experiments were conducted using the PyTorch 1.13.1 framework on a workstation equipped with an Intel Xeon Gold 5220R CPU and an NVIDIA A4000 GPU. The input images had a size of 224 × 224 × 3. During data augmentation, the Adam optimizer was used with betas of 0.5 and 0.999. The learning rates of the generator and discriminator were set to 0.0001 and 0.0002, respectively. The generator takes a 100-dimensional noise vector sampled from a standard normal distribution as input. The batch size was set to 64, and the number of epochs was 1600. For the recognition stage, the Adam optimizer was used with betas of 0.9 and 0.999, a learning rate of 0.001, a batch size of 64, and 120 training epochs. During the training phase, the dataset was divided into training set and validation set in a ratio of 8:2. Additionally, the test set consists of 200 images from a single category that are independent of the training and validation sets. This configuration ensured stable convergence and maintained high performance for both the generative and classification models. For parameter settings, all comparison models retained their original architectures and parameters. The complete training configurations are shown in Table 1 and Table 2. All GAN-based models were trained from scratch using identical training protocols, including the same batch size, number of epochs, optimizer settings, and data augmentation strategies. Fixed learning rates were adopted for both the generator and discriminator without learning-rate decay or dropout regularization to ensure stable adversarial optimization. All baseline classification models were trained using the same data augmentation pipeline and learning-rate scheduling strategy to ensure fair comparison.

### 3.3. Evaluation Metrics

To comprehensively assess the performance of the proposed RGSGAN–MACRNet framework, both generation quality metrics and classification performance metrics were employed.

For the generative stage, two quantitative measures were used to evaluate the quality and diversity of synthesized images, namely the Inception Score (IS) and the Fréchet Inception Distance (FID). The IS evaluates both the confidence and the diversity of generated images by using the conditional label distribution p(y|x) predicted by the Inception-V3 network:

(7) $$IS=exp\left(E_{x∼p_{g}(x)}\left[D_{KL}(p(y|x)||p(y))\right]\right)$$

where $D_{KL}$ denotes the Kullback–Leibler divergence, p(y|x) is the conditional label probability of the generated image x, and $p(y)=\int p(y|x)p_{g}(x)\;dx$ represents the marginal class distribution. A higher IS value indicates that the generated samples are both classifiable and diverse, implying better visual quality and variety.

The FID measures the statistical distance between the feature distributions of real and generated samples in the Inception feature space. Assuming that the features follow a multivariate Gaussian distribution, the FID is defined as:

(8) $$FID=∥\mu_{r}-\mu_{g}{∥}_{2}^{2}+\mathrm{Tr}\left(\Sigma_{r}+\Sigma_{g}-2(\Sigma_{r}\Sigma_{g}{)}^{\frac{1}{2}}\right)$$

where $\left(\mu_{r},\Sigma_{r}\right)$ and $\left(\mu_{g},\Sigma_{g}\right)$ are the mean and covariance of real and generated image features, respectively. A lower FID indicates that the generated images are more similar to real samples in terms of both distribution and perceptual quality. In this work, both IS and FID were computed on identical quantities of generated and real samples under identical preprocessing to ensure fair comparison.

For the computation of the FID, the official pretrained Inception-V3 network provided by the PyTorch library was employed, adhering to the standard configuration for FID evaluation widely adopted in GAN assessment studies. Feature representations were extracted from the network’s final global average pooling layer (pool3), yielding 2048-dimensional feature vectors for each image. No fine-tuning of the Inception-V3 model was performed to maintain the integrity of the standard evaluation pipeline. Both real and generated images were resized to 224 × 224 pixels and processed using an identical normalization scheme prior to being fed into the Inception-V3 network, with no additional image enhancement, post-processing, or model adjustments applied. This resizing strategy follows commonly used FID implementations and does not affect the relative comparison between different models. To avoid evaluation bias and ensure fairness, the number of generated samples was matched to that of real samples, specifically, 6000 generated images were randomly sampled to align with the size and class distribution of the real GrainSpace dataset. The same feature extraction and preprocessing pipeline was consistently applied across all models. For FID score calculation, the mean vectors and covariance matrices of the extracted features were computed separately for real and generated samples. All experiments were conducted under uniform preprocessing conditions with fixed random seeds to guarantee the reproducibility and consistency of results across different model comparisons.

For the classification stage, we adopted several standard metrics, including accuracy (Acc), precision (Pre), recall (Rec), and F1-score (F1). Let the true positive (TP), true negative (TN), false positive (FP), and false negative (FN) values denote the number of samples in each prediction category. These indicators are defined as follows:

(9) $$Accuracy=\frac{TP+TN}{TP+TN+FP+FN}$$

(10) $$Precision=\frac{TP}{TP+FP}$$

(11) $$Recall=\frac{TP}{TP+FN}$$

(12) $$F1=\frac{2\times Precision\times Recall}{Precision+Recall}$$

Accuracy reflects the overall proportion of correctly classified samples. Precision and recall describe the ability of the model to correctly identify positive samples, and the F1-score provides a harmonic balance between them, particularly useful when class distributions are imbalanced. In addition, a confusion matrix was employed to visually represent the prediction outcomes for each kernel category, providing intuitive insight into inter-class misclassification patterns.

To further evaluate the computational efficiency of the model, we reported the number of parameters (Params) and the floating-point operations (FLOPs). These two indicators, respectively, reflect the memory footprint and the computational cost of the model, which are critical for assessing its suitability for real-time or embedded deployment. A lower parameter count and FLOP value indicate a more lightweight and efficient network. The FLOPs and parameters were computed using the torch summary library to ensure accurate and consistent measurements across all models.

By jointly analyzing the generation metrics (IS, FID), classification metrics (Accuracy, Precision, Recall, F1), and efficiency metrics (Params, FLOPs), a comprehensive and objective evaluation of the proposed RGSGAN–MACRNet framework is achieved. These indicators collectively reflect the data realism, recognition accuracy, and computational efficiency, thereby validating the effectiveness and practicality of the proposed method.

### 3.4. Evaluation of Data Augmentation Effectiveness

To evaluate the effectiveness of the proposed RGSGAN in generating high-quality images, two quantitative metrics were used: FID and IS. FID measures the statistical distance between the distributions of authentic and generated images, with lower values indicating greater realism and diversity. IS assesses image clarity and distinguishability, with higher values indicating better quality. To ensure the validity and fairness of the experiments, all models were trained from scratch under the same experimental conditions, without any pretraining. When using the MNIST dataset, the batch size was set to 64 and the number of training epochs to 100 for all models. For the GrainSpace dataset, the batch size was fixed at 64 and the number of training epochs was set to 1600 for all models. Model weights are saved at intervals of 10 epochs, and the weights corresponding to the minimum FID score are ultimately selected as the optimal model weights. All models were trained from scratch under identical conditions, with the same number of generated samples as real images used for both IS and FID evaluations. All input images were uniformly resized to 224 × 224. A random seed was set for each experiment, and the random seeds of PyTorch, numpy, and Python were fixed to ensure reproducibility. The official Inception-v3 pretrained model was employed to compute IS and FID. For FID calculation, 6000 generated samples (consistent with the distribution of real samples) were used, with comparisons conducted against the feature statistics of the entire real dataset. Table 3 and Table 4 present performance comparisons among different models on the MNIST and GrainSpace datasets, respectively.

**Figure 8:**
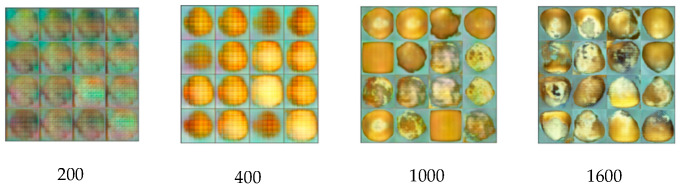
Generated imperfect kernel samples produced by RGSGAN at different training epochs.

On the MNIST dataset, the proposed RGSGAN achieved an FID score of 29.37, substantially lower than those of WGAN (39.22), DCGAN (41.89), and DCGAN-GP (38.14), demonstrating a closer match between generated and real sample distributions. On the GrainSpace dataset, RGSGAN outperforms baseline models in generation performance. Baseline models such as WGAN, CGAN, LSGAN, and DCGAN achieved ISs of 6.0, 6.9, 5.7, and 5.3, with corresponding FID values of 285.5, 244.6, 225.0, and 313.1, while ACGAN improved to IS 7.7 and FID 288.5. RGSGAN reached an IS of 8.5 and an FID of 148.1, showing substantial gains in both diversity and quality. Against optimized strategies including Residual, SCSA, and WGAN-GP combinations, RGSGAN consistently outperformed models such as DCGAN+ Residual+ WGAN-GP (IS 6.9, FID 190.2) and DCGAN+ SCSA+ WGAN-GP (IS 7.8, FID 175.9). Specifically, RGSGAN improved IS by 3.2 and reduced FID by 165.0 compared to the baseline DCGAN, highlighting the effectiveness of its spatial–channel attention mechanism and stable training design. In summary, the proposed RGSGAN achieves the best generative performance on the GrainSpace dataset, with IS and FID metrics significantly outperforming those of comparison models. An FID of 148 indicates that RGSGAN effectively approximates the real data distribution. This is further supported by stable improvements in downstream classification, demonstrating that the generated images provide meaningful and high-quality feature diversity, confirming the model’s excellent performance for GrainSpace image generation tasks. As shown in Figure 8, the visual quality of generated imperfect kernel samples progressively improved with increasing training epochs (200, 400, 1000, 1600). The generated images accurately captured features such as mold, breakage, and dark spots without mode collapse, validating the stable training behavior of RGSGAN.

**Figure 9:**
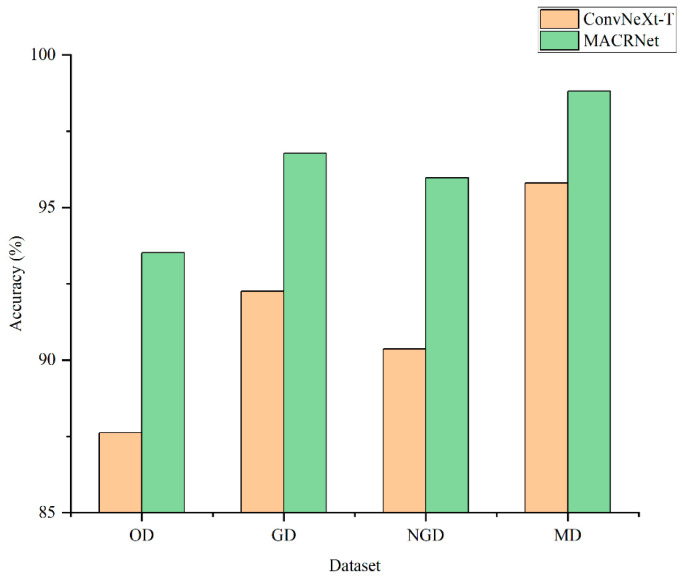
Recognition accuracies of ConvNeXt-T and MACRNet trained using four datasets: Original Dataset; Generative Dataset; Non-Generative Dataset; Mixed Dataset.

To further validate the enhancement effect of RGSGAN, both ConvNeXt-T and MACRNet were trained and tested on four datasets: OD (Original Dataset), GD (Generative Dataset), NGD (Non-Generative Dataset), and MD (Mixed Dataset). As illustrated in Figure 9, for ConvNeXt-T, the mixed dataset achieved an accuracy of 95.801%, improving by 8.176 percentage points compared with the original dataset; generative and non-generative augmentations improved accuracy by 4.631% and 2.742%, respectively. For MACRNet, accuracy on the mixed dataset reached 98.813%, improving by 5.292% over the original dataset, while generative and non-generative augmentation yielded gains of 3.255% and 2.451%, respectively. The mixed augmentation strategy thus outperformed both single-type augmentations, suggesting that generative and non-generative data augmentations provide complementary benefits in the feature space, mitigating overfitting and improving generalization. Notably, the weaker ConvNeXt-T network showed larger improvements, indicating that greater data diversity can narrow the performance gap among models.

### 3.5. Comparison of Classification Performance

**Figure 10:**
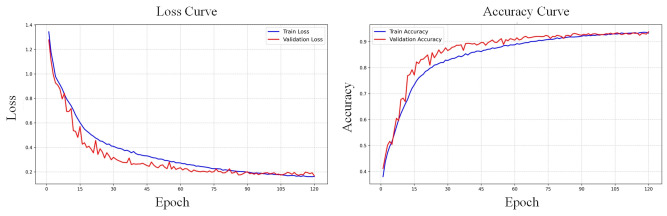
Training and validation accuracy and loss curves of the MACRNet model.

**Figure 11:**
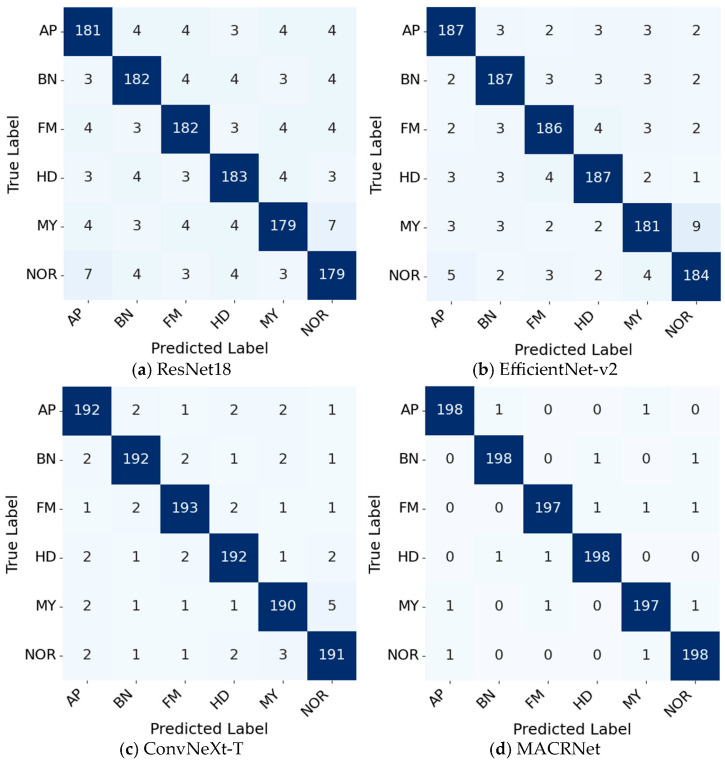
Confusion matrices of different models.

To validate the classification effectiveness of the proposed MACRNet, multiple mainstream CNN architectures were compared. All baseline networks were trained from scratch under identical hyperparameters (a batch size of 64, 120 epochs) as MACRNet to ensure fair comparison. No pre-trained weights were used. Figure 10 shows the training and validation accuracy and loss curves of MACRNet, while Figure 11 presents confusion matrices for four representative models. According to the confusion matrix results, ResNet18, EfficientNet-V2, and ConvNeXt-T exhibit a noticeable tendency to misclassify moldy kernels as normal kernels. This misjudgment occurs with a significantly higher probability than other confusion cases. The underlying reason lies in their feature extraction mechanisms. Traditional convolutional architectures primarily focus on capturing global contour and shape information of corn kernels. Consequently, these models tend to emphasize macroscopic features such as kernel outline, overall texture, and brightness distribution, while neglecting subtle local defects. For moldy kernels, the key discriminative cues often lie in small-scale mold spots and slight color variations scattered over the surface. These fine-grained local textures are difficult for standard convolutional models to capture effectively, leading to a tendency to confuse them with normal kernels that have intact outlines. In contrast, the proposed MACRNet integrates multi-scale asymmetric convolutional residual blocks, allowing it to simultaneously extract global shape information and local fine-grained details. As a result, MACRNet achieves not only higher overall accuracy but also more balanced misclassification behavior across all categories, demonstrating its stronger feature discrimination and generalization capabilities.

To ensure the reliability and stability of the results, all experiments were conducted three times with different random seeds, and the average values were reported. This repeated-training strategy provides a statistically more stable estimate of model performance under limited-sample conditions. As summarized in Table 5, MACRNet achieved a classification accuracy of 98.813%, outperforming ResNet18, EfficientNet-v2, ConvNeXt-T, and ConvNeXt-v2 by 8.3%, 6.16%, 3.01%, and 4.09%, respectively. In terms of macro-averaged precision (macro-P), recall (macro-R), and F1-score (macro-F1), MACRNet also surpassed other competitive models such as ResNet50, InceptionNeXt, Swin, ViT, VAN, and RepViT. Table 6 further compares the computational complexity and parameter counts of all models. MACRNet required only 4.542 G FLOPs and 8.446 M parameters, far fewer than most baseline networks, while achieving the highest accuracy. This confirms that MACRNet achieves a superior balance between efficiency and precision, making it highly suitable for engineering and real-time applications.

**Figure 12:**
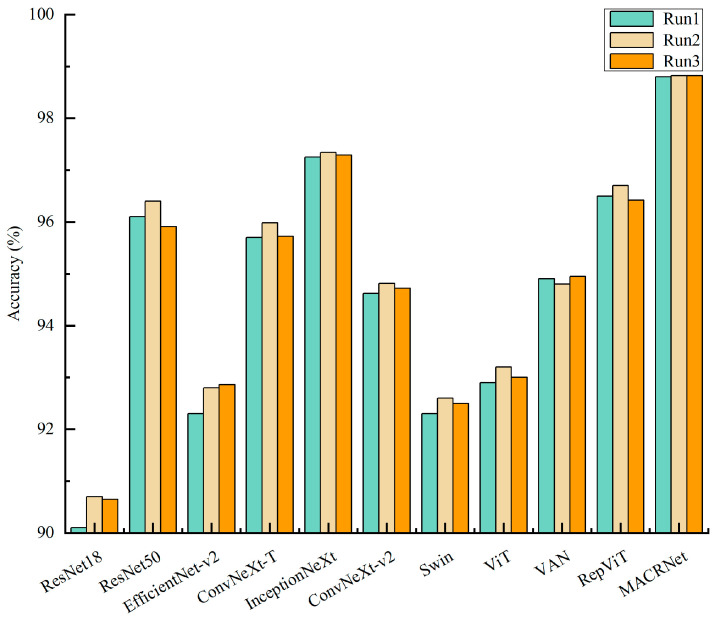
Classification accuracy of different models across three independent runs.

To enhance the statistical rigor of the experiments, each model was trained inde pendently three times. The accuracy values obtained from these three independent runs are summarized in Figure 12, and the corresponding statistical metrics, including the mean (Mean), standard deviation (Std), standard error (Std Error), and the 95% confidence interval (95% CI), are reported in Table 7. Confidence intervals were computed using a t-distribution with two degrees of freedom (t = 4.303), enabling a reliable assessment of the performance variability and stability of each model. The results in Figure 12 and Table 6 show that all models exhibit relatively narrow 95% confidence intervals, indicating stable training processes and strong reproducibility across runs. Notably, the proposed MACRNet exhibits the smallest performance fluctuation (Std = 0.012) and a remarkably tight 95% confidence interval ranging from 98.79% to 98.84%. These findings further demonstrate the robustness, stability, and reproducibility of MACRNet for imperfect maize kernel recognition.

**Figure 13:**
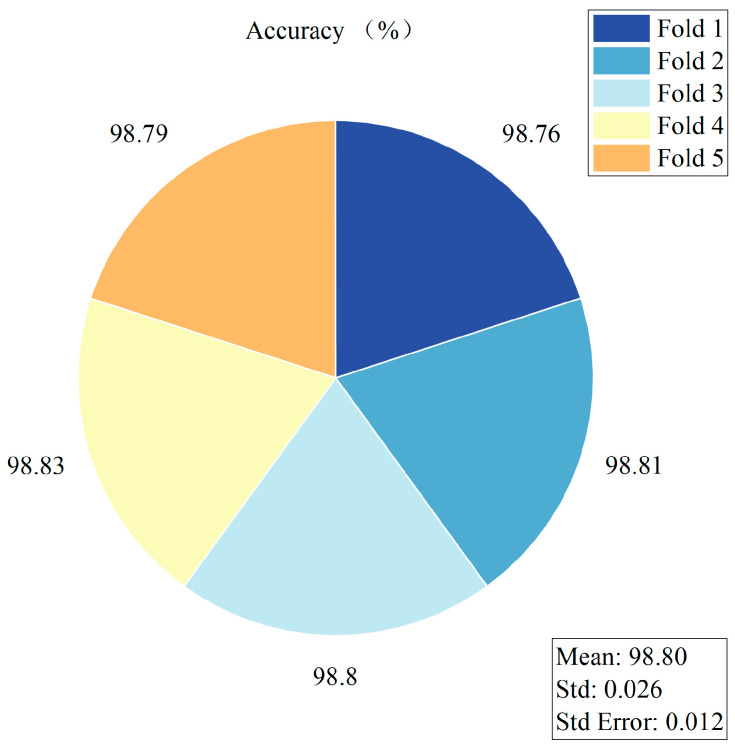
MACRNet accuracy statistics in 5-fold cross-validation.

To further evaluate the generalization ability and stability of the proposed MACRNet, we conducted a 5-fold cross-validation (5-fold CV). Each fold maintained an identical sample distribution and consistent hyperparameter settings, and the full training pipeline was executed to convergence within each fold. The test accuracy of each fold, along with the cross-fold mean (Mean), standard deviation (Std), and standard error (Std Error), is visualized in Figure 13. MACRNet achieved an average accuracy of 98.80% across the five folds, with a standard deviation of 0.026, demonstrating its strong stability and robustness. Moreover, these 5-fold CV results are highly consistent with the performance observed in the three independent training experiments, further confirming the reproducibility and reliability of the experimental conclusions.

### 3.6. Ablation Studies

To verify the effectiveness of different convolutional branches in the MACR module, ablation experiments were conducted by sequentially removing individual branches and comparing their effects on model parameters, computation cost, and classification accuracy. As illustrated in Table 8, the complete MACRNet achieved the highest accuracy. Removing the 3 × 1/1 × 3 branches resulted in a 6.7% drop in accuracy, underscoring their crucial role in capturing fine-grained texture details. Similarly, removing the 5 × 1/1 × 5 and 9 × 1/1 × 9 branches resulted in notable performance degradation, highlighting the importance of medium- and large-scale convolutions for capturing global structural information. By contrast, removing the 7 × 1/1 × 7 branches caused only a minor accuracy drop, suggesting partial redundancy in receptive fields. Therefore, in resource-constrained scenarios, the 7 × 1/1 × 7 branches can be simplified or merged to reduce computational cost while retaining other key branches for optimal performance. These findings validate the rationality of the MACR design and the effectiveness of its multi-scale structure. Overall, the proposed network achieves a superior trade-off between computational efficiency and recognition precision, providing a practical solution for imperfect recognition of sample-size-limited corn kernels.

## 4. Discussion

In this paper, we proposed the RGSGAN–MACRNet framework to address imperfect kernel recognition under limited-sample conditions. The proposed method achieved an accuracy of 98.813% on the enhanced dataset, with a maximum improvement of 8.3% over baseline models. These performance gains primarily stem from two components: the Spatial–Channel Synergistic Attention module, which improves the fidelity and structural consistency of synthetic samples by emphasizing defect-related regions, and the asymmetric multi-branch design of MACRNet, where smaller receptive fields capture subtle crack and mold textures while larger branches model overall kernel morphology. Together, these designs demonstrate that integrating generative augmentation with multi-scale asymmetric convolution is an effective strategy for fine-grained agricultural image recognition.

Compared with related studies, the proposed method exhibits clear methodological and performance advantages. In [49], a single CNN with limited data enhancement constrains the modeling of fine-grained kernel defects, whereas the multi-scale feature fusion mechanism in MACRNet effectively enhances sensitivity to subtle defect patterns. Unlike previous work [45], which relies on costly hyperspectral imaging systems, the proposed method achieves superior performance using only standard RGB images, enabling a more practical and cost-effective deployment. Although previous studies [46] report 97.79% accuracy on a large-scale dataset of 31,000 images, their performance strongly depends on abundant samples and classifier-focused optimization, while the proposed approach achieves a higher accuracy of 98.813% with only 6000 images, demonstrating stronger robustness under limited-sample conditions. Moreover, compared with the SMOTE-based strategy in [48], which lacks defect-specific structural realism, the proposed RGSGAN generates more targeted and realistic defect samples, leading to more effective data augmentation and improved recognition performance.

Despite these promising results, several limitations remain regarding generalizability. The dataset consists of RGB corn kernel images acquired under controlled laboratory conditions. Although the framework is conceptually applicable to other crops, such as wheat, rice, soybeans, and peanuts, variations in texture, defect morphology, and spectral properties may require task-specific adjustments to the attention modules or multi-scale convolutional design. In addition, real-world field environments introduce challenges not reflected in laboratory data, including variable illumination, dust, kernel overlap, camera inconsistencies, and cultivar diversity, which may cause domain shifts and affect model stability. While generative augmentation mitigates data scarcity, synthetic samples derived from controlled settings may not fully represent real-world variability.

Future work will focus on improving robustness and scalability under practical conditions. First, cross-crop and cross-season generalization should be systematically evaluated by incorporating field-acquired samples from multiple crop species and imaging devices. Second, domain adaptation, semi-supervised, and self-supervised learning strategies should be explored to reduce reliance on large labeled datasets and improve resilience to unseen variations. Finally, model efficiency can be enhanced through pruning, quantization, and edge-oriented deployment, facilitating reliable integration into industrial grain-sorting systems.

## 5. Conclusions

To overcome the challenge of achieving high-accuracy recognition of imperfect corn kernels under sample-size-limited conditions, this research proposed a novel and highly efficient recognition framework integrating RGSGAN and MACRNet. The proposed method initially combines RGSGAN with traditional data augmentation strategies to expand the dataset, and subsequently employs MACRNet for efficient feature extraction and classification, thereby significantly enhancing recognition performance under limited-sample conditions.

Specifically, RGSGAN integrates residual structures and a spatial–channel synergistic attention mechanism into the generator while incorporating the Wasserstein distance with a gradient penalty term. These innovations jointly improve the authenticity and diversity of generated samples, ensure stable and controllable optimization, and provide high-quality synthetic data to fundamentally address sample scarcity. RGSGAN achieves superior performance on both FID and IS metrics compared with conventional DCGAN, WGAN, LSGAN, CGAN, and ACGAN models, generating images with higher clarity, richer texture details, and enhanced structural consistency. Meanwhile, MACRNet, built with multi-scale asymmetric convolutional residual modules, enables collaborative fusion of local and global features via a multi-branch convolutional architecture. This design substantially reduces parameters and computation cost while maintaining strong classification capability, thereby offering an efficient alternative to complex deep models. Experimental results indicate that the proposed method achieves 98.813% accuracy on the enhanced dataset, surpassing ResNet18, EfficientNet-v2, ConvNeXt-T, and ConvNeXt-v2 by 8.3%, 6.16%, 3.01%, and 4.09%, respectively, and outperforms the model trained on the original dataset by 5.29%. Further ablation studies verify that the multi-scale asymmetric convolution branches within the MACR module complement each other in learning both fine-grained defects and global semantic cues, and their synergistic integration is pivotal to accuracy improvement. In summary, the proposed RGSGAN-MACRNet framework offers a new paradigm for tackling intelligent grain-quality assessment under data-limited scenarios. It substantially improves recognition accuracy and stability for imperfect corn kernels, and provides a generalizable and scalable solution with strong theoretical significance and practical application potential for future agricultural visual inspection and industrial deployment.

## Figures and Tables

**Table 1:** Training configurations of different models.

Model	Optimizer	Epochs	Batch Size	Loss Function	LR Schedule	Data Processing	Dropout Rate
ResNet18 ResNet50	SGD Initial Lr: 1 × 10^−2^ momentum: 0.9 Weight Decay: 1 × 10^−4^	120	64	Cross- Entropy	Cosine annealing from initial Lr to 1 × 10^−6^	Resize: (224, 224) Normalization: mean = [0.647, 0.638, 0.448] std = [0.177, 0.192, 0.209] Transformations: random resize cropping horizontal flipping	None
EfficientNet-v2	RMSProp Initial Lr: 4 × 10^−3^ momentum: 0.9 Weight Decay: 1 × 10^−4^						0.2
ViT	Adam Initial Lr: 3 × 10^−3^ betas: (0.9, 0.999) Weight Decay: 1 × 10^−4^						0.2
Swin	AdamW Initial Lr: 1 × 10^−3^ betas: (0.9, 0.999) Weight Decay: 1 × 10^−4^						0.2
ConvNeXt-T InceptionNeXt ConvNeXt-v2 RepViT VAN	AdamW Initial Lr: 4 × 10^−3^ betas: (0.9, 0.999) Weight Decay: 1 × 10^−4^						None
MACRNet	Adam Initial Lr: 1 × 10^−3^ betas: (0.9, 0.999) Weight Decay: 1 × 10^−4^						0.2

**Table 2:** Training configurations of different GANs.

Model	Data Processing	Generator Optimizer	Discriminator Optimizer	Batch Size	Epochs	Loss Function
DCGAN	Resize: (224, 224) Normalization: mean = [0.647, 0.638, 0.448] std = [0.177, 0.192, 0.209]	Adam Lr: 1 × 10^−4^ betas: (0.5, 0.999) Weight Decay: 0	Adam Lr: 2 × 10^−4^ betas: (0.5, 0.999) Weight Decay: 0	64	1600	BCE
DCGAN-GP						WGAN-GP
WGAN						Wasserstein
CGAN						BCE
LSGAN						Least Squares
ACGAN						BCE + Aux loss
RGSGAN						WGAN-GP

**Table 3:** Performance comparison of different models on MNIST.

Model	FID
WGAN	39.22
DCGAN	41.89
DCGAN-GP	38.14
**RGSGAN (ours)**	**29.37**

**Table 4:** Performance comparison of different models and optimization strategies on GrainSpace.

Model	IS	FID
WGAN	6.0	285.5
CGAN	6.9	244.6
LSGAN	5.7	225.0
ACGAN	7.7	288.5
DCGAN	5.3	313.1
DCGAN -GP	6.3	273.6
DCGAN+ Residual	5.6	296.5
DCGAN+ SCSA	6.8	251.1
DCGAN+ Residual+ WGAN-GP	6.9	190.2
DCGAN+ Residual+ SCSA	6.2	230.7
DCGAN+ SCSA+ WGAN-GP	7.8	175.9
**RGSGAN (ours)**	**8.5**	**148.1**

**Table 5:** Average performance comparison among different models.

Model	Macro-P (%)	Macro-R (%)	Macro-F1 (%)	Accuracy (%)
ResNet18	89.702	90.382	90.041	90.482
ResNet50	95.597	96.257	95.926	96.137
EfficientNet-v2	91.734	92.604	92.167	92.654
ConvNeXt-T	95.501	96.201	95.850	95.801
InceptionNeXt	97.143	97.513	97.328	97.293
ConvNeXt-v2	94.115	94.815	94.464	94.715
Swin	91.588	92.268	91.927	92.468
ViT	92.579	93.179	92.878	93.029
VAN	94.632	94.982	94.807	94.882
RepViT	95.861	96.591	96.225	96.541
**MACRNet (ours)**	**98.693**	**99.013**	**98.853**	**98.813**

**Table 6:** Comparison of FLOPs and parameters across different models.

Model	FLOPs (G)	Parameters (M)	Accuracy (%)
ResNet18	1.826	11.72	90.482
ResNet50	3.848	22.43	96.137
EfficientNet-v2	2.723	19.38	92.654
ConvNeXt-T	4.157	26.53	95.801
InceptionNeXt	3.910	24.57	97.293
ConvNeXt-v2	4.149	26.51	94.715
Swin	4.147	25.71	92.468
ViT	4.359	26.98	93.029
VAN	4.859	25.76	94.882
RepViT	5.984	29.33	96.541
**MACRNet** **(ours)**	**4.542**	**8.446**	**98.813**

**Table 7:** Statistical accuracy metrics of different models over three runs.

Model	Mean	Std	Std Error	95% CI
ResNet18	90.482	0.327	0.189	90.09–90.87
ResNet50	96.137	0.249	0.144	95.52–96.75
EfficientNet-v2	92.654	0.309	0.178	92.00–93.31
ConvNeXt-T	95.801	0.153	0.088	95.42–96.17
InceptionNeXt	97.293	0.045	0.026	97.18–97.41
ConvNeXt-v2	94.715	0.097	0.056	94.48–94.95
Swin	92.468	0.153	0.088	92.09–92.85
ViT	93.029	0.153	0.088	92.66–93.40
VAN	94.882	0.076	0.044	94.69–95.07
RepViT	96.541	0.148	0.085	96.17–96.91
**MACRNet (ours)**	**98.813**	**0.012**	**0.007**	**98.79–98.84**

**Table 8:** Performance comparison of branch removal ablation experiments.

Model	FLOPs (G)	Parameters (M)	Accuracy (%)
remove the 3 × 1/1 × 3 branch	4.544	8.448	92.136
remove the 5 × 1/1 × 5 branch	4.541	8.445	94.222
remove the 7 × 1/1 × 7 branch	4.537	8.441	96.421
remove the 9 × 1/1 × 9 branch	4.533	8.446	93.745
**MACRNet (ours)**	**4.542**	**8.446**	**98.813**

## Data Availability

The original contributions presented in the study are included in the article; further inquiries can be directed to the corresponding author.
